# Carbon price prediction based on decomposition technique and extreme gradient boosting optimized by the grey wolf optimizer algorithm

**DOI:** 10.1038/s41598-023-45524-2

**Published:** 2023-10-27

**Authors:** Mengdan Feng, Yonghui Duan, Xiang Wang, Jingyi Zhang, Lanlan Ma

**Affiliations:** 1 Department of Civil Engineering, Henan University of Technology, No. 100, Lianhua Street, Gaoxin District, Zhengzhou, 450001 China; 2 Department of Civil Engineering, Zhengzhou University of Aeronautics, No. 15, Wenyuan West Road, Zhengdong New District, Zhengzhou, 450015 China

**Keywords:** Climate-change impacts, Projection and prediction

## Abstract

It is essential to predict carbon prices precisely in order to reduce CO_2_ emissions and mitigate global warming. As a solution to the limitations of a single machine learning model that has insufficient forecasting capability in the carbon price prediction problem, a carbon price prediction model (GWO–XGBOOST–CEEMDAN) based on the combination of grey wolf optimizer (GWO), extreme gradient boosting (XGBOOST), and complete ensemble empirical mode decomposition with adaptive noise (CEEMDAN) is put forward in this paper. First, a random forest (RF) method is employed to screen the primary carbon price indicators and determine the main influencing factors. Second, the GWO–XGBOOST model is established, and the GWO algorithm is utilized to optimize the XGBOOST model parameters. Finally, the residual series of the GWO–XGBOOST model are decomposed and corrected using the CEEMDAN method to produce the GWO–XGBOOST–CEEMDAN model. Three carbon emission trading markets, Guangdong, Hubei, and Fujian, were experimentally predicted to verify the model’s validity. Based on the experimental results, it has been demonstrated that the proposed hybrid model has enhanced prediction precision compared to the comparison model, providing an effective experimental method for the prediction of future carbon prices.

## Introduction

Climate change has evolved into a formidable menace to the survival of humanity in the twenty-first century. Greenhouse gases are considered a major factor contributing to global warming^1^. To cope with the global warming crisis, the international community has actively reduced carbon emissions by formulating climate policies and other measures. Among them, the European Emissions Trading System (EU-ETS) was implemented in 2005, reducing carbon emissions and energy consumption^2^. Furthermore, China plays a significant role in international climate protection as one of the top carbon emitters worldwide. China has implemented eight carbon trading pilots in various regions, namely Beijing (2013), Shanghai (2013), Guangdong (2013), Tianjin (2013), Shenzhen (2013), Chongqing (2014), Hubei (2014), and Fujian (2016), in order to reduce global emissions^3^.

Carbon trading has emerged as an emerging financial industry. A carbon price reflects fluctuations in supply and demand for carbon energy within the carbon emissions market, where carbon energy can be traded as a commodity^4^. Because of the uncertainty of the internal mechanism and external factors, carbon prices demonstrate nonlinear and non-stationary features^5,6^. The risks associated with carbon trading are greater than those associated with traditional financial products. Accurate carbon price forecasting not only helps governments grasp the changes in market conditions and make reliable decisions, but also helps enterprises and investors grasp the characteristics of carbon prices. This will make sensible resource allocations and realize the value-added of carbon assets. As a result, it is crucial to establish a system that is stable and effective for the research of carbon prices.

In accordance with the previous literature review, carbon price research can be categorized into two classifications: models based on historical data^7–9^ and models based on influencing factors^10–13^.

Grounded on historical data, carbon price forecasting methods can be classified into three categories: statistical and econometric methods, artificial intelligence (AI), and integration methods.

In the past, statistical and econometric methods were extensively employed for forecasting carbon prices as classical time series forecasting methods. Main statistical methods are the autoregressive integrated moving average model (ARIMA)^14^, generalized autoregressive conditional heteroskedasticity model (GARCH)^15^, gray model (GM)^16^, etc. For instance, Carolina et al. (2013) employed an ARIMA model in order to forecast carbon prices, ultimately achieving more accurate predictive outcomes^17^. According to Dutta (2018), an exponential GARCH model was used for forecasting carbon price volatility, and outliers were processed to improve accuracy^18^. Under the assumption of linearity, the statistical and econometric methods perform well for short-term forecasting, but when forecasting nonlinear, non-stationary time series of carbon prices, the prediction accuracy is not satisfactory^19^.

As a result, AI that does not require linear assumptions is broadly utilized across different sectors. For example, credit risk prediction^20^, disease treatment^21^, and traffic congestion^22,23^. For carbon price prediction, least squares support vector machines (LSSVM) and artificial neural networks (ANN) are commonly used. Using 1074 daily carbon price results, Atsalakis (2016) developed a neural network (NN) model to predict time series. ANN was found to be the most effective method for predicting carbon prices based on the final results^24^. Zhu et al. (2016) introduced an adaptive multiscale integrated learning approach grounded in LSSVM to effectively capture the non-stationary and non-linear attributes of carbon prices. The findings demonstrated that their proposed model surpassed the performance of the ARIMA and GARCH models^25^. Despite the fact that AI exceeds traditional statistical models in forecasting non-linear and non-stationary data, a single AI model fails to possess sufficient forecasting stability and does not meet researchers’ expectations for accurate carbon price predictions across different markets^26^.

Given the constraints of conventional statistical approaches in handling non-stationary feature data and the shortcomings of a single AI model, experts have started to focus on researching integrated methods to boost data analysis and forecasting precision. A number of decomposition methods have been proposed based on different theoretical foundations, including the wavelet transform (WT)^27^, variational mode decomposition (VMD)^28^, and ensemble empirical mode decomposition (EEMD)^29^. E et al. (2019) realized that carbon valence has nonlinear and nonstationary properties. To address this issue, they combined VMD with a gated recurrent unit (GRU) to predict carbon prices’ future trends. Experimental results confirmed its validity and reliability^30^. Jinpei Liu et al. (2019) employed empirical mode decomposition (EMD) and a reconstruction algorithm to transform the original data into three subseries of varying frequencies. Subsequently, they individually analyzed these three types of data using ARIMA, partial least squares (PLS), and NN methods. The findings demonstrated the superior predictive performance of the model^31^. Using EEMD to preprocess the data, Zhou et al. (2018) constructed different combinations of models to identify different frequencies. The existing hybrid models, although they enhance carbon price prediction accuracy, have drawbacks^32^. For example, existing hybrid models usually have model subseries obtained from decomposition without considering noise. This can reduce prediction accuracy and efficiency^29^.

Carbon prices are impacted by a combination of historical data and external factors. The existing literature primarily utilizes carbon price time series data for the modeling process. However, the dynamics of carbon trading prices are influenced by various factors, including energy factors, macroeconomic factors, and industry structures^11^. In general, since external factors can be analyzed, carbon price forecasting built upon multiple influencing factors is important for carbon market research. Therefore, carbon price prediction models for influencing factors are favored by scholars. Using oil, coal, and natural gas prices as the basis, Tsai and Kuo (2013) devised an ant-based radial basis function network (ARBFN) model for carbon price prediction. The inclusion of multiple influencing factors in carbon price forecasting models can indeed pose challenges due to the potential for error accumulation. When considering multiple factors, the complexity of the model increases, and uncertainties associated with each factor can accumulate throughout the forecasting process^33^.

Reviewing previous studies, we identify potential research gaps in the prediction of carbon prices. One is that most carbon price forecasting models rely only on past carbon price data series. They ignore the impact of external factors on the carbon market. This limitation may result in models that do not adequately take into account the full range of market conditions when forecasting carbon prices. Second, most current carbon price forecasting models fail to fully explore and utilize other useful information. Useful information means that after the model prediction, there are still a large number of nonlinear residual sequences, which are not random walks^34^ and still contain carbon price information. Ignoring the residual series leads to the potential problem of incomplete information in predicting carbon prices. To this end, it is of particular importance that the above issues are addressed and a new perspective on carbon price forecasting is proposed.

In order to bridge these gaps, this study first established the index system of influencing factors of carbon price and selected indicators by the random forest method to find out the main factors affecting carbon price, so as to improve the prediction accuracy of the model. Secondly, XGBOOST is used to establish the carbon price prediction model. Meanwhile, with the objective of avoiding the prediction error caused by the parameter setting of the XGBOOST model, GWO is used to find the optimization of the model parameters. To increase the precision of model predictions, the residual series of XGBOOST predictions is corrected using the CEEMDAN method, and a combined GWO–XGBOOST–CEEMDAN model is derived. The contributions can be summarized as follows:

1. In the majority of prior studies, carbon price forecasts relied on historical time series data on carbon prices. This ignores the effects of multiple factors when predicting carbon prices, so there are limits to the information that can be provided and the extent to which carbon markets can be managed. In this study, multiple influencing factors are considered in conducting carbon price forecasts with the aim of addressing the problem of carbon price forecasting. In order to develop a richer indicator system that is more appropriate to China’s national conditions, the carbon price time series data as well as the various influencing factors are treated as candidate input features for carbon price modeling.
2. In this study, Partial Autocorrelation Function (PACF) and Random Forest (RF) are introduced as feature selection methods to build carbon price prediction models in accordance with numerous influencing factors and reduce the influence of redundant information between features. A significant improvement has been made in the model’s prediction performance.
3. Most previous hybrid models first decompose the data and then perform carbon price prediction studies. However, this study adopts a different approach by first predicting carbon prices and then decomposing the residual series. After the carbon price information is predicted by the strong master model, the useful information of the residual sequence is difficult to obtain, so the CEEMDAN algorithm is used to further process the residual information and decompose it into modal information that is easy to extract and a sequence that is more difficult to extract. This is to dig deeper into residual effective information. According to the experiment, carbon price prediction is more accurate and practical than most previous studies. The method of prediction and then decomposition offers innovative thought for carbon price prediction research, and it will serve as a strong reference in the future.

## Algorithm introduction

### Feature election

#### Random forest

After the initial selection of 11 metrics, feature screening is performed next. It can enhance the model’s ability to generalize, reduce the risk of overfitting, reduce the computational complexity of the model, etc. Common feature selection methods are Gray correlation, Pearson correlation coefficient, and random forests (RF). Gray correlation and Pearson correlation coefficient are both linear relationship-based methods, while RF can handle more complex nonlinear relationships. This means RF can select features in a wide range of situations. As a result, in this paper, the RF method is used for screening carbon price primary indicator systems.

Based on the results of the RF method, the primary features are ranked in terms of importance and then selected. Consider a sample size of $A$ and a feature dimension of $m$. Provide a set of training samples $\left\{({x}_{1},{y}_{1}),\cdots ,({x}_{N},{y}_{N})\right\}$ and create a self-help sample set ${C}_{t}$ of size $A$; ${K}_{t}$ is obtained by classification and regression tree (CART) on ${C}_{t}$ ; Taking a random sample of ${m}_{try}=\sqrt{m}$ features from each tree and selecting the most significant ${m}_{try}$ features for node splitting; Analyzing whether $t$ satisfies $t\le ntree$ until the loop is not exited, and then generating $G=Uniform\left(\left\{{K}_{t}\right\}\right)$.

In the calculation of feature importance, the Gini Index is used as a segmentation function to calculate "Gini Importance" as the degree of importance of a feature. This can be expressed as follows:

1 $$Gini(C)=1-{{\sum }_{i=1}^{\left|E\right|}\left[{F}_{i}\right]}^{2}$$

$C$ represents the sample set;${F}_{i}$ represents the probability of belonging to the $ith$ class in the sample set $C$ ; There are a number of sample classes in $E$ . The Gini index of the sample set $C$ is defined when feature $G$ is known.

2 $$Giniindex(C,G)={\sum }_{H=1}^{H}\frac{\left|{C}^{H}\right|}{C}Gini({C}^{H})$$

$H$ represents the number of features $G$ values, i.e., $C$ is divided into $H$ subsets according to the feature $G$ values $\left\{{C}^{1},{C}^{2},...{C}^{H}\right\}$, and the samples within each subset are of the same feature $G$ value. $G$ feature that has the smallest Gini index after division is considered to be the optimal feature in the selection process.

#### Partial auto-correlation function

PACF is a statistical tool for time series analysis that helps determine the relationship between each observation in a time series and its lag values. Its function is to recognize the order of the AR (Autoregressive) model in a time series, i.e., how many lags need to be considered in that model. The PACF model actually adjusts the autocorrelation function (ACF) by eliminating the part already explained by the previous lags so that the remaining part more accurately reflects the relationship between the observations and the lags at the current moment. $({X}_{t},{X}_{t+v}|{X}_{t+1}|,\cdot \cdot \cdot ,{X}_{\left(t+v-1\right)}$ represents the conditional correlation between ${X}_{t}$ and ${X}_{t+v}$ after removing the effects of the intervening variables ${X}_{t+1},\cdot \cdot \cdot ,{X}_{\left(t+v-1\right)}$, i.e., the partial autocorrelation between ${X}_{t}$ and ${X}_{t+v}$.

### CEEMDAN model

Empirical mode decomposition (EMD) is to decompose the nonlinear and non-stationary raw data into inherent mode functions (IMF_S_) with various fluctuation scales. However, due to the intermission of the raw data, mode confusion is easy to occur. This will affect the decomposition effect. Wu^35^ proposed an ensemble empirical mode decomposition (EEMD) method by adding a certain degree of Gaussian white noise to the original data for repeated decomposition. Although the mode overlap phenomenon can be effectively solved, residual white noise still exists in the component of the eigenmode function derived by this method, resulting in low reconstruction accuracy. Building upon this, Torres^36^ moved to the complete ensemble empirical mode decomposition with adaptive noise (CEEMDAN) method, which addresses the issue of significant reconstruction errors in the EEMD method by introducing adaptive white noise at each stage. Therefore, in this essay, the CEEMDAN method is used to forecast each component of the eigenmode function and the trend term separately.

CEEMDAN can be broken down as follows:

*Step 1* As a result of adding a Gaussian white noise sequence to the residual sequence, an updated sequence with noise is obtained:

3 $$\overline{{y}_{i}}\left(t\right)=y\left(t\right)+\sigma {n}_{i}\left(t\right),i=\mathrm{1,2}...N$$

where $y\left(t\right)$ is the residual sequence, and $\overline{{y}_{i}}\left(t\right)$ is the new sequence with the addition of Gaussian white noise; ${n}_{i}\left(t\right)$ denotes the white noise added to the residual data; σ is the adaptive coefficient.

*Step 2* EMD decomposition is performed on the new sequence with white noise added to obtain N modal components, and the first modal component of CEEMDAN is obtained by the overall averaging of the N modal components as follows:

4 $$im{f}_{1}\left(t\right)=\frac{1}{N}{\sum }_{i=1}^{N}im{f}_{1i}\left(t\right)$$

At this point, ${R}_{1}\left(t\right)$ is the residual component.

5 $$ {\text{R}}_{1} \left( {\text{t}} \right) = \overline{{{\text{y}}_{{\text{i}}} }} \left( {\text{t}} \right) - {{imf}}_{1}^{\mathrm{^{\prime}}} \left( {{t}} \right) $$

*Step 3* The adaptive white noise sequence $\sigma {n}_{i}\left(t\right)$ is added to ${R}_{1}\left(t\right)$ to form a new sequence ${R}_{1}\left(t\right)+\sigma {E}_{1}\left({n}_{i}\left(t\right)\right)$ with noise, where ${E}_{j}\left(\cdot \right)$ is the jth eigenmodal component obtained after EMD decomposition. At this point, the EMD decomposition is performed on the new sequence and averaged to obtain the second modal component and the residual component as follows:

6 $$ im{f}_{2} \left( {{t}} \right) = \frac{1}{{\text{N}}}\mathop \sum \limits_{{{\text{i}} = 1}}^{{\text{N}}} {\text{E}}_{1} \left( {{\text{R}}_{1} \left( {\text{t}} \right) + {\upsigma }_{1} {\text{E}}_{1} \left( {{\text{n}}_{{\text{i}}} \left( {\text{t}} \right)} \right)} \right) $$

7 $${R}_{2}\left(t\right)={R}_{1}\left(t\right)-im{{f}_{2}}^{\mathrm{^{\prime}}}\left(t\right)$$

*Step 4* Repeat the above three steps to obtain the (j + 1)th modal component and the jth residual component:

8 $$im{f}_{j+1}\left(t\right)=\frac{1}{N}{\sum }_{i=1}^{N}{E}_{1}\left({R}_{j}\left(t\right)+{\sigma }_{j}{E}_{j}\left({n}_{i}\left(t\right)\right)\right)$$

9 $${R}_{j}\left(t\right)={R}_{j-1}\left(t\right)-im{{f}_{j}}^{\mathrm{^{\prime}}}\left(t\right)$$

*Step 5* Repeat the above steps until the CEEMDAN can no longer be decomposed by EMD. Finally, the original sequence $\mathrm{y}(\mathrm{t})$ is decomposed into multiple eigenmodal components and a trend component.

10 $$ {\text{y}}\left( {\text{t}} \right) = {{imf}}\left( {\text{t}} \right) + {\text{R}}_{{{\text{es}}}} \left( {\text{t}} \right) $$

After CEEMDAN has decomposed the residual series, the GWO–XGBOOST model is applied to each eigenfunction component. The final residual forecast is derived by linearly combining the results of each component.

### XGBOOST model

Extreme gradient boosting (XGBOOST) was developed by Chen et al.^37^ in 2016, which integrates a linear scale solver with a categorical regression tree learning algorithm. The model combines models with low prediction accuracy through certain strategies. The purpose of this is to construct an integrated model that is more accurate in terms of prediction. During the model training process, XGBOOST optimizes the boosting process. Each iteration generates an updated decision tree to fit the residuals generated in the previous iteration. XGBOOST can continuously improve its prediction accuracy and generalization capacity through iterative optimization. While traditional gradient boosting decision tree (GBDT) methods utilize only first-order derivatives, XGBOOST does a second-order Taylor expansion of the loss function, controls model complexity by introducing regularization terms to avoid overfitting problems, and employs a more refined evaluation approach when splitting nodes to better capture the nonlinear relationships between features. In recent years, the XGBOOST model has shown superior performance in financial risk control, medical health, natural language processing, and other fields. This model is based on the following mathematical principles:

An integration model for the definition tree can be described as follows:

11 $${\widehat{y}}_{i}={\sum }_{m=1}^{M}{f}_{m}({x}_{i}),{f}_{m}\in F$$

where ${\widehat{y}}_{i}$ is the prediction value; $M$ is the number of decision trees; $F$ is the tree selection space; ${x}_{i}$ is the first $i$ input feature.

XGBOOST’s loss function is as follows:

12 $$Q={\sum }_{i=1}^{n}l({y}_{i},{\widehat{y}}_{i})+{\sum }_{m=1}^{M}\theta \left({f}_{m}\right)$$

The first part of the function is the prediction error between the predicted value and the real training value of the XGBOOST model, and the second part represents the complexity of the tree, which is mainly used to control the regularization of the model complexity:

13 $$\theta ({f}_{m})=\gamma T+\frac{1}{2}\tau {\Vert \omega \Vert }^{2}$$

where $\gamma$ and $\tau$ are penalty factors.

By adding an incremental function ${f}_{t}\left({x}_{i}\right)$ to Eq. (13), the value of the loss function is minimized. Then the objective function of the $t$ th time is

14 $${Q}_{\left(t\right)}={\sum }_{i=1}^{n}l({y}_{i},{\widehat{y}}_{i})+{\sum }_{m=1}^{M}\theta \left({f}_{m}\right)={\sum }_{i=1}^{n}l\left({y}_{i},{{\widehat{y}}_{i}}^{t-1}+{f}_{t}\left({x}_{i}\right)\right)+\theta \left({f}_{t}\right)$$

The second-order Taylor expansion of Eq. (15) is used to approximate the objective function, and the set of samples in each child of the $j$ tree is defined as ${I}_{j}=\left\{i\left|q\left({x}_{i}=j\right)\right.\right\}$. At this point the ${Q}_{\left(t\right)}$ can be approximated as

15 $${Q}_{\left(t\right)}\cong \sum_{j=1}^{T}\left[\left({\sum }_{i\in {I}_{j}}{g}_{i}\right){\omega }_{j}+(1/2)\left({\sum }_{i\in {I}_{j}}{h}_{i}+\tau \right){{\omega }_{j}}^{2}\right]+\gamma T$$

where ${g}_{i}={\partial }_{{{\widehat{y}}_{i}}^{t-1}}l\left({y}_{i},{{\widehat{y}}_{i}}^{t-1}\right)$ is the first order derivative of the loss function; ${h}_{i}={{\partial }^{2}}_{{{\widehat{y}}_{i}}^{t-1}}l\left({y}_{i},{{\widehat{y}}_{i}}^{t-1}\right)$ is the second order derivative of the loss function. Defining ${G}_{i}=\sum i\in {I}_{j}{g}_{i}$, ${H}_{i}={\sum }_{i\in {I}_{j}}{h}_{i}$ then we have:

16 $${Q}_{\left(t\right)}\cong {\sum }_{j=1}^{T}\left[{G}_{j}{\omega }_{j}+(1/2)\left({H}_{j}+\tau \right){{\omega }_{j}}^{2}\right]+\gamma T$$

The partial derivative of $\omega$ yields

17 $${\omega }_{j}=-{G}_{j}/{(H}_{j}+\tau )$$

By incorporating weights into the objective function, we get

18 $${Q}_{\left(t\right)}\cong -(1/2){\sum }_{j=1}^{T}{{G}_{j}}^{2}/({H}_{j}+\tau )+\gamma T$$

A large portion of the model’s performance is determined by parameter selection during the training process of the XGBOOST model. There are 23 hyperparameters in the XGBOOST algorithm, mainly divided into general parameters for macroscopic function control, booster parameters for booster detail control, and learning target parameters for training target control. The GWO–XGBOOST combinatorial model combines the three hyperparameters that have a significant impact on the performance of XGBOOST (learning_rate, n_estimators, and max_depth) as the position vector of the head wolf $\alpha$ in the GWO algorithm and continuously updates them through the iterations of the GWO algorithm to continuously find the optimal position until the global optimal position is output as the final parameter of the XGBOOST model.

### GWO model

A pack intelligence optimization algorithm, the grey wolf optimizer (GWO), based on the predatory behavior of grey wolves, was proposed by Mirjalili et al.^38^ in 2014, inspired by the predatory behavior of grey wolves. The optimization process of the GWO algorithm can be analogized to the hunting behavior of the gray wolf pack. Among them, *α*, $\beta$, and $\delta$ wolves with the highest social level in each generation of the population act as the leaders of the gray wolf pack. A predator searches, encircles, and attacks prey to achieve its optimization goal. GWO has strong global convergence ability, robustness, and fewer parameters to adjust, and is now used in many fields for optimization problems.

Firstly, the mathematical definition of how a wolf pack searches for and surrounds its prey is as follows:

19 $$A=\left|B\cdot {F}_{p}\left(t\right)-F\left(t\right)\right|$$

20 $$F\left(t+1\right)={F}_{p}\left(t\right)-C\cdot A$$

21 $$c=2-2D/E$$

22 $$C=2c\cdot {r}_{1}-c$$

23 $$B=2\cdot {r}_{2}$$

where $F\left(t\right)$ is the position of the prey after the $t$ th iteration; ${F}_{P}\left(t\right)$ is the position of the gray wolf at the $t$ iteration; $A$ is the distance between the gray wolf and the prey; $F\left(t+1\right)$ is the update of the position of the gray wolf; $C$ and $B$ are the coefficient vectors;$c$ is the convergence factor whose value decreases linearly from 2 to 0 with the number of iterations, $D$ is the number of previous iterations, and $E$ is the maximum number of iterations; $r_{1}$ and $r_{2}$ are the random numbers between [0,1].

Secondly, the prey is finally determined by constantly updating the positions of the three optimal wolves *α*, $\beta$, and $\delta$. The mathematical definition of the hunting process of the gray wolf pack is

24 $${A}_{\alpha }=\left|{B}_{1}\cdot {F}_{\alpha }\left(t\right)-F\left(t\right)\right|$$

25 $${A}_{\beta }=\left|{B}_{2}\cdot {F}_{\beta }\left(t\right)-F\left(t\right)\right|$$

26 $${A}_{\delta }=\left|{B}_{3}\cdot {F}_{\delta }\left(t\right)-F\left(t\right)\right|$$

27 $${F}_{1}\left(t+1\right)={F}_{\alpha }\left(t\right)-{C}_{1}\cdot {A}_{\alpha }$$

28 $${F}_{2}\left(t+1\right)={F}_{\beta }\left(t\right)-{C}_{2}\cdot {A}_{\beta }$$

29 $${F}_{3}\left(t+1\right)={F}_{\delta }\left(t\right)-{C}_{3}\cdot {A}_{\delta }$$

30 $$F\left(t+1\right)=({F}_{1}\left(t+1\right)+{F}_{2}\left(t+1\right)+{F}_{3}\left(t+1\right))/3$$

where ${F}_{\alpha }\left(t\right)$, ${F}_{\beta }\left(t\right)$ and ${F}_{\delta }\left(t\right)$ are the positions of $\alpha$, $\beta$ and $\delta$ wolves when the population is iterated to generation t; $F\left(t\right)$ is the position of individual gray wolves in generation t; ${C}_{1}$ and ${B}_{1}$, ${C}_{2}$ and ${B}_{2}$, ${C}_{3}$ and ${B}_{3}$ are the coefficient vectors of $\alpha$, $\beta$ and $\delta$ wolves, respectively; ${F}_{1}\left(t+1\right)$,${F}_{2}\left(t+1\right)$ and ${F}_{3}\left(t+1\right)$ are the positions of $\alpha$, $\beta$ and $\delta$ wolves after $\left(t+1\right)$ iterations, respectively; $F\left(t+1\right)$ is the position of the next generation of gray wolves.

### GWO–XGBOOST–CEEMDAN model

**Figure 1:**
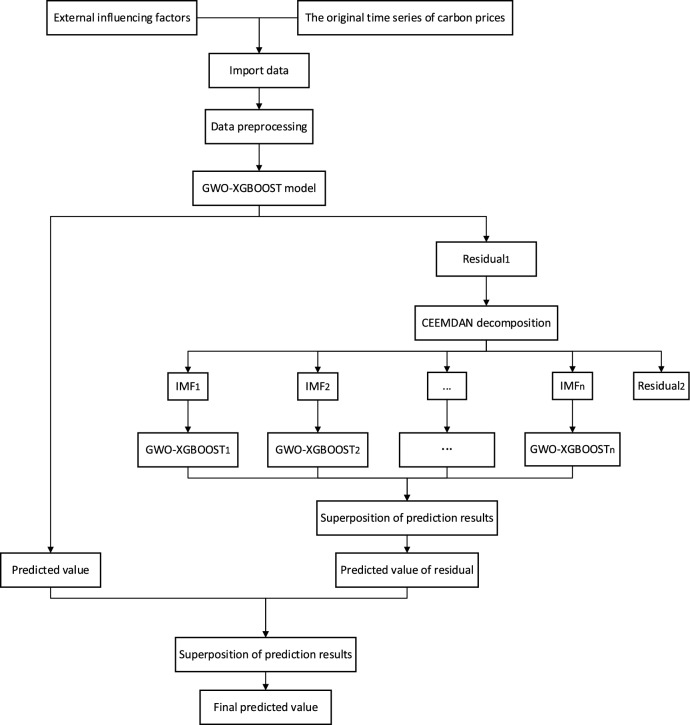
GWO–XGBOOST–CEEMDAN model prediction process.

To improve carbon price prediction, we propose to combine the CEEMDAN, XGBOOST, and GWO models to build the GWO–XGBOOST–CEEMDAN model. The general idea is as follows: First, the GWO–XGBOOST model is established, and the GWO algorithm is used for optimizing the parameters of the XGBOOST model. Secondly, the CEEMDAN method is applied to decompose the residual series of the GWO–XGBOOST model to establish the GWO–XGBOOST–CEEMDAN hybrid model. Finally, the predicted values and the accumulated values of the residual predictions are summed up to get the final prediction results of the model. Figure 1 illustrates the specific process.

## Data description

### Data source

Accurate carbon price forecasts smooth investment decisions and maintain carbon market stability. There are big differences between China’s carbon trading pilots. The Hubei carbon trading market is the only carbon trading market in central China^12^. In addition, the Guangdong carbon market was officially launched in 2013, setting five first places in China’s carbon market trading^39^. Fujian is the first ecological civilization demonstration zone in China. The carbon market is aligned with the overall idea of the national carbon market, and it is the first pilot to adopt carbon verification standards and guidelines issued by the state. In particular, the data direct reporting system is completely consistent with the national system under construction standards, and the construction starting point is high^40,41^. To sum up, this paper chooses Guangdong, Hubei, and Fujian carbon trading markets as research objects. In this paper, we collect data on the three carbon markets from the Choice financial terminal and the Wind database. The selected carbon prices take into account public holidays, differences in trading hours, and missing values of variables at home and abroad. In the above data, the Bohai Sea Power Coal Price Index and Natural Gas Market Quotation are weekly and ten-day data, and Eviews software is used to convert them into daily data. A hybrid model is evaluated by using 80% of the data for training and 20% for testing. The carbon price information for the three trading markets is shown in Table 1, and Table 2 presents descriptive statistics for each indicator.

**Table 1:** Carbon trading market data information.

Market	Dataset	Training set	Test set	Training date	Test date	Time nodes
Guangdong	703	563	140	2020/4/3–2022/7/14	2022/7/15–2023/2/3	2020/4/3–2023/2/3
Hubei	750	601	149	2020/1/2–2022/8/24	2022/8/25–2023/4/10	2020/1/2–2023/4/10
Fujian	699	560	139	2018/1/2–2022/9/16	2022/9/19–2023/5/5	2018/1/2–2023/5/5

**Table 2:** Descriptive statistics of carbon price data.

Index name	Mean	Std	Var	Min	25%	50%	75%	Max
Research object I: Guangdong carbon market—related variables								
Y	50.95700	21.67600	469.86900	27.04000	28.48000	42.61000	77.02000	95.26000
X1.1	3105.00500	283.22600	80,216.69900	2247.32000	2856.65000	3019.20000	3229.08000	3544.69000
X1.2	31,804.61600	3344.51100	11,185,756.23000	21,052.53000	29,883.79000	32,861.34000	34,451.23000	36,799.65000
X1.3	3938.82600	489.80400	239,908.31400	2488.65000	3655.04000	3963.51000	4348.87000	4793.54000
X1.4	4579.15800	513.71300	263,900.86800	3508.70000	4098.71000	4712.31000	4964.77000	5807.72000
X2.2	662.55000	88.60400	7850.71000	525.35000	573.14000	683.08000	735.00000	850.94000
X2.3	5188.03000	1821.86100	3,319,179.05200	2490.57000	3491.55000	5445.17000	6740.67000	8615.91000
X3.1	56.53500	23.80500	566.68300	17.86000	30.81000	57.70000	79.72000	97.59000
X4.3	62.03100	29.04700	843.73200	13.00000	41.00000	53.00000	76.00000	201.00000
Research object II: Hubei carbon market—related variables								
Y	37.91793	9.21166	84.85500	24.49000	28.96750	38.26000	47.44000	61.48000
X1.2	31,662.85910	3400.11127	11,560,756.66000	18,591.93000	29,869.74000	32,630.30500	34,293.65000	36,799.65000
X1.3	3910.49480	496.30847	246,322.10100	2237.40000	3653.15250	3944.67500	4275.00000	4793.54000
X1.4	4527.37730	518.30981	268,645.06100	3508.70000	4046.36750	4628.22500	4939.75750	5807.72000
X2.1	72.60910	23.53668	553.97500	19.50000	51.43750	74.10000	87.70250	129.47000
X2.2	662.95280	85.60991	7328.91900	508.96000	576.48740	673.27550	735.00000	863.67000
X2.3	5135.90040	1746.30901	3,049,595.17500	2488.45000	3614.98900	5246.80680	6604.83080	8676.97000
X3.1	57.30340	24.86076	618.05800	15.45000	29.74500	58.40500	80.95500	97.67000
X4.3	63.57550	31.84088	1013.84200	13.00000	42.00000	63.23500	73.25000	239.00000
Research object III: Fujian carbon market—related variables								
Y	20.74330	6.73573	2,712,776.15600	2542.36000	16.25000	19.19000	25.28000	38.12000
X1.1	2774.70790	356.36149	126,993.51400	1975.77000	2481.34000	2835.87000	3032.92000	3544.69000
X1.2	29,673.45940	3707.38569	13,744,708.68000	22,445.37000	26,112.53000	29,673.46000	33,156.41000	36,432.22000
X1.3	3535.71570	627.95655	394,329.42300	2416.62000	2884.43000	3535.72000	4017.82000	4791.19000
X2.1	76.34070	19.25121	370.60900	39.54000	64.40000	74.78000	87.23000	124.37000
X2.2	650.75130	86.31098	7449.58600	531.04000	575.71000	595.10000	734.00000	884.03000
X2.3	5113.61820	1647.05074	2,712,776.15600	2542.36000	3678.41000	4684.76000	6602.58000	8557.84000
X3.1	49.74140	30.12951	907.78700	7.62000	24.90000	32.02000	80.84000	97.67000
X4.3	41.86680	14.44742	208.72800	13.00000	31.00000	41.87000	49.00000	126.00000

### ADF inspection

The ADF (Augmented Dickey–Fuller) test was proposed by economists David Dickey and Wayne Fuller in 1979. The test is a statistical method used to determine whether the time series data has a unit root (or the root of the series), i.e., to verify whether the data has smoothness. The ADF test gives a Guangdong *p*-value of 0.912439, a Hubei *p*-value of 0.638039, and a Fujian *p*-value of 0.988874, which are greater than the usually chosen significance level (e.g., 0.05 or 0.01). Therefore, the original hypothesis cannot be rejected; that is, the historical carbon price data of the three carbon markets is not stationary. In short, it is not possible to use traditional econometric methods for experiments, and an integrated learning approach is used for the prediction study of non-stationary time series of carbon prices.

### Data pre-processing

The factors often have different magnitudes and units of magnitude. It is crucial to pretreat the data to be limited to [0, 1] to remove the adverse effects caused by odd sample data and make the data comparable.

31 $${Z}^{*}=\frac{Z-{Z}_{min}}{{Z}_{max-{Z}_{min}}}$$

where ${Z}^{*}$ represents the normalized value of the data; $Z$ is the input data, and ${Z}_{min}$ and ${Z}_{max}$ represent the minimum and maximum values of the input data, respectively.

### Four aspects that affect the price of carbon

Carbon prices are impacted by a number of factors. This paper builds primary indicators of carbon price influencing factors from four aspects: macroeconomics, energy prices, international carbon markets, and weather conditions. The detailed classification and secondary quantification of each indicator level are shown in Table 3.

**Table 3:** Primary indicators of carbon price impact factors.

Primary indicators	Secondary indicators	Factor symbols	Serial number	Literature sources
Macroeconomics	Shanghai Stock Exchange Industrial Index	SHZQ	X1.1	^10,64^
	Dow Jones Industrial Average	DQS	X1.2	^10^
	S&P 500 Index	BP500	X1.3	^10,64^
	CSI300 Index	CSI300	X1.4	^65,10^
Energy prices	Brent Crude Oil CFD	CFD	X2.1	^66,47,10^
	Bohai Sea Power Coal Price Index	HBH	X2.2	^10^
	Natural Gas Market Offer	TRQ	X2.3	^10,47^
International carbon markets	EU Carbon Emission Allowances	EUA	X3.1	^66,11^
	Maximum Temperature	ZGQW	X4.1	^47^
Weather conditions	Minimum Temperature	ZDQW	X4.2	^47^
	Air Quality Index	AQI	X4.3	^47,10,13^

#### Macroeconomics

The macroeconomy directly determines the boom in the carbon market^42^. The macroeconomic situation, specifically the advancement of the industrial economy, is the most representative of CO_2_ emissions, which will affect the price of carbon trading^43^. Guo Fuchun says that when macroeconomic conditions are favorable, production and business activities become active, and the carbon trading price will enter a relatively stable operation. In contrast, when the economy slows down, the carbon trading price will fluctuate sharply^44^. Meanwhile, carbon prices also reflect a country’s economic growth to a certain extent^45^. In addition, China’s carbon trading market is developing and maturing. It covers a broad spectrum of industries, including chemicals, construction materials, steel, non-ferrous metals, and various other sectors. Therefore, the rapid economic development and extension of China’s carbon market highlight the significance of carbon pricing mechanisms^46^.

#### Energy prices

It is often considered that energy is the factor that has the greatest impact on carbon prices^47^. With this in mind, researchers have been empirically searching for the drivers of carbon prices and understanding their future value through adequate predictive analysis. Early studies have identified energy prices as one of the indicators of the main influencing factors of carbon prices^26,48–50^. For example, crude oil^51^ and natural gas prices^52^. The majority of these studies have found that energy prices have a significant impact on carbon prices. However, investment strategies need to focus not only on the impact of external factors on carbon prices but also on the predictability of future returns. Consequently, numerous recent studies have concentrated on the predictability of carbon prices’ future values^53,54^. In conclusion, it is valuable to study the influence of energy prices on carbon prices both now and in the future.

#### International carbon markets

Foreign carbon markets influence China’s carbon prices^11^. On the one hand, Chinese carbon markets are still in the development stage. In contrast, foreign carbon markets have been established for a longer period of time and are relatively well-established. China’s carbon market will, to a certain extent, refer to foreign carbon markets when setting carbon emission quotas. For instance, the EUA price acts as the primary reference point in the global carbon trading market, significantly shaping carbon emission allowances and, consequently, carbon prices^55^. Specifically, EUA, certified emission reductions (CER), and other similar products are used to reference the fulfillment of carbon reduction obligations^29^. On the other hand, the disparity in economic development between China and other nations can result in variations in carbon market pricing. If China’s carbon market is priced low, transnational companies will speculate heavily in the Chinese carbon market to buy a large number of carbon emission rights, thus adding to the demand for carbon emission rights in the Chinese market and driving up the Chinese carbon price until it reaches parity with the international carbon price. Furthermore, at the macro level, an increase in carbon emissions reduces foreign direct investment, which affects the trading of carbon allowances and indirectly causes price volatility^10^. As of now, China’s carbon market is not yet in line with international standards. Consequently, the investor base remains relatively modest in size. Nevertheless, once the two are connected, the issue of speculation is expected to escalate. Hence, foreign carbon prices will have a dual effect: they will inform the establishment of carbon prices in China and potentially drive up the carbon price in the country through speculative activities^11^.

#### Weather conditions

Global warming is becoming more severe and the primary cause of this issue can be attributed to greenhouse gas emissions, especially CO_2_^56^. Climate change can affect carbon price volatility through multiple channels. Earlier studies have shown that climate change can alter fossil energy consumption and thus affect carbon price fluctuations^57–60^. In the past few years, researchers have mainly addressed the significance of climate change on carbon prices from different perspectives. From a production standpoint, when temperatures become excessively high or low, residents resort to cooling or heating equipment, resulting in a temporary upswing in energy consumption and subsequent CO_2_ emissions. In addition, from a business perspective, extreme weather and catastrophic events are exposing new energy companies to a significant physical risk, leading to changes in the energy mix and having a considerable impact on carbon prices^61,62^. To be more precise, the generation of environmentally friendly energy sources like wind, solar, and hydropower is strongly influenced by various weather factors, including temperature, precipitation, and humidity^63^. Therefore, it is very important to consider climate change when predicting carbon prices^46^.

## Experimental results and discussion

### Evaluation indicators

In this study, five common metrics are used, as shown in Table 4. The larger the R^2^ and the smaller the remaining indicators, the better the predictive performance of the model.

**Table 4:** Evaluation indicators.

Indicators	Definition	Formula
MSE	Mean square error	$MSE=\frac{1}{n}{{\sum }_{i=1}^{n}\left({y}_{i}-\widehat{{y}_{i}}\right)}^{2}$
MAE	Mean absolute error	$MAE=\frac{1}{n}{\sum }_{i=1}^{n}\left|{y}_{i}-\widehat{{y}_{i}}\right|$
RMSPE	Root mean square percentage error	$RMSPE=\sqrt{\frac{1}{n}{\sum }_{i=1}^{n}{\left|\frac{{y}_{i}-\widehat{{y}_{i}}}{{y}_{i}}\right|}^{2}}*100\%$
MAPE	Mean absolute percentage error	$MAPE=\frac{1}{n}{\sum }_{i=1}^{n}\left|\frac{{y}_{i}-\widehat{{y}_{i}}}{{y}_{i}}\right|*100\%$
${R}^{2}$	Coefficient of determination	${R}^{2}=1-\frac{S{S}_{residual}}{S{S}_{total}}$

### Experiment one: comparison of this paper’s model with different benchmark models

#### Algorithm table

To demonstrate the superiority of the prediction performance of the proposed GWO–XGBOOST–CEEMDAN model in practical applications, four other different benchmark models are first set up for comparison in this paper, namely GBDT, XGBOOST, GWO–XGBOOST, and GWO–XGBOOST–EEMD. As indicated in Table 5.

**Table 5:** Comparison of different models.

Models	Decomposition technology	Parameter optimization
GBDT		
XGBOOST		
GWO–XGBOOST		√
GWO–XGBOOST–EEMD	√	√
PROPOSED MODEL	√	√

#### Model parameter setting

To validate the prediction accuracy of the GWO–XGBOOST–CEEMDAN model, various comparison algorithms are utilized to evaluate forecasting performance. For the prediction of the base model, the GBDT and XGBOOST models are selected to compare and analyze the prediction effect of GWO–XGBOOST. For the prediction of the combined residual correction model, the EEMD and CEEMDAN methods are used to compare and decompose the residual sequences generated by GWO–XGBOOST. The parameter settings of each model are shown in Table 6. According to the actual situation of the three carbon trading markets, the parameters of each model are adjusted as shown in Table 7, and the remaining parameters are set by default in Python.

**Table 6:** Parameters of each model.

Models	Adjustment parameters
GBDT	n_estimators; min_samples_leaf; learning_rate; max_depth; min_samples_split
XGBOOST	n_estimators; colsample_bytree; learning_rate; max_depth
GWO–XGBOOST	n_estimators; colsample_bytree; learning_rate; max_depth; subsample

**Table 7:** Model parameter settings.

Models	Guangdong parameters	Hubei parameters	Fujian parameters
GBDT	(100,2,0.1,8,4)	(30,9,0.1,20,20)	(15,8,0.1,20,20)
XGBOOST	(100,0.5,0.1,10)	(100,0.2,0.1,18)	(100,0.3,0.1,20)
GWO–XGBOOST	(37.5037,0.3176,0.2457, 56.2732, 0.5770)	(31.1208, 0.4177, 0.0441, 40.7127, 0.1817)	(11.3251, 0.1224, 0.0683, 19.2370, 0.5652)

#### Screening analysis of carbon price influencing factors

**Figure 2:**
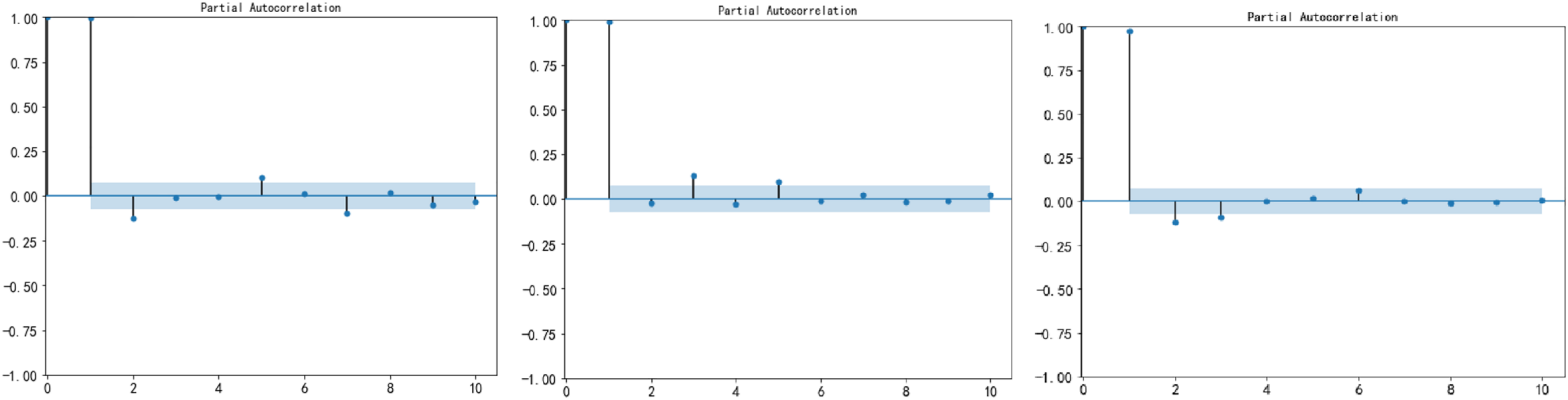
PACF results for carbon price data.

This study considers both the selection of historical carbon price variables and the identification of external influences in three carbon trading markets. More specifically, for the purpose of examining the correlation between historical carbon price variables and the carbon price data, we use PACF in order to identify the relevant input data characteristics for forecasting. Figure 2 shows the PACF results for Guangdong, Hubei, and Fujian. This analysis reveals a notable fourth-order autocorrelation in the carbon price data of Guangdong, whereas both Hubei and Fujian demonstrate a third-order autocorrelation. Xi is the output feature; {Xi-1, Xi-2, Xi-5, Xi-7} are the input historical variables for the Guangdong dataset; {Xi-1, Xi-3, Xi-5} are the input historical variables for the Hubei dataset; and {Xi-1, Xi-2, Xi-3} are the input history variables for the Fujian dataset.

**Figure 3:**
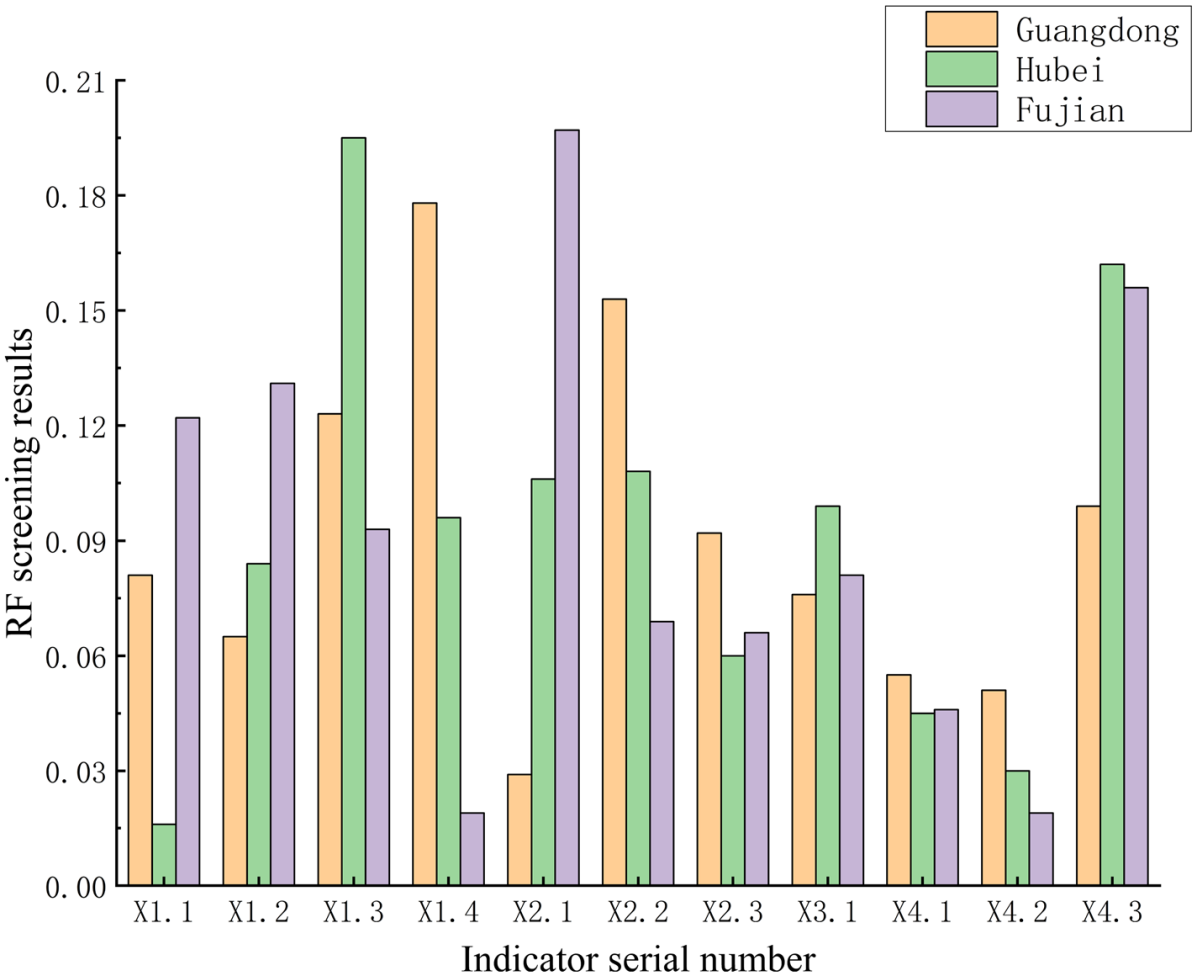
Random Forest screening results.

Furthermore, with the intention of identifying the main external influences on carbon prices, this paper selects 11 primary carbon price indicators and uses random forest to rank the importance of the indicators for the purpose of indicator screening, as shown in Fig. 3. The screened carbon price indicators are shown in Table 8. The input variables for the Guangdong data set are {Xi-1, Xi-2, Xi-5, Xi-7, X1.1, X1.2, X1.3, X1.4, X2.2, X2.3, X3.1, X4.3}, and the input variables for the Hubei data set are {Xi-1, Xi-3, Xi-5, X1.2, X1.3, X1.4, X2.1, X2.2, X2.3, X3.1, X4.3}, and the input variables for the Fujian dataset are {Xi-1, Xi-2, Xi-3, X1.1, X1.2, X1.3, X2.1, X2.2, X2.3, X3.1, X4.3}.

**Table 8:** Carbon price finalization indicators.

	Guangdong	Hubei	Fujian
Macroeconomics	Shanghai Stock Exchange Industrial Index (X1.1)	Dow Jones Industrial Average (X1.2)	Shanghai Stock Exchange Industrial Index (X1.1)
	Dow Jones Industrial Average (X1.2)	S&P 500 Index (X1.3)	Dow Jones Industrial Average (X1.2)
	S&P 500 Index (X1.3)	CSI300 Index (X1.4)	S&P 500 Index (X1.3)
	CSI300 Index (X1.4)		
Energy prices	Bohai Sea Power Coal Price Index (X2.2)	Brent Crude Oil CFD (X2.1)	Brent Crude Oil CFD (X2.1)
	Natural Gas Market Offer (X2.3)	Bohai Sea Power Coal Price Index (X2.2)	Bohai Sea Power Coal Price Index (X2.2)
		Natural Gas Market Quotes (X2.3)	Natural Gas Market Quotes (X2.3)
International carbon markets	EU Carbon Emission Allowances (X3.1)	EU Carbon Emission Allowances (X3.1)	EU Carbon Emission Allowances (X3.1)
Weather conditions	Air Quality Index (X4.3)	Air Quality Index (X4.3)	Air Quality Index (X4.3)

#### Carbon price forecast results I

**Figure 4:**
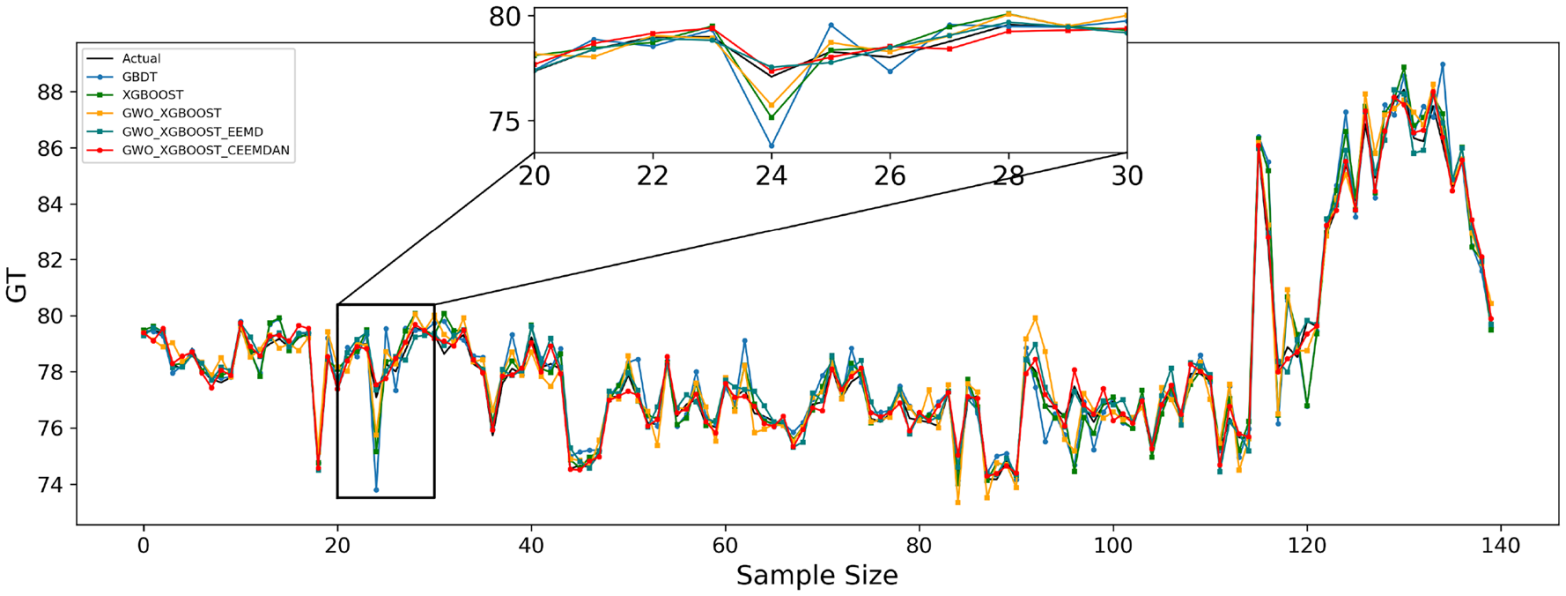
Guangdong model prediction effect.

**Figure 5:**
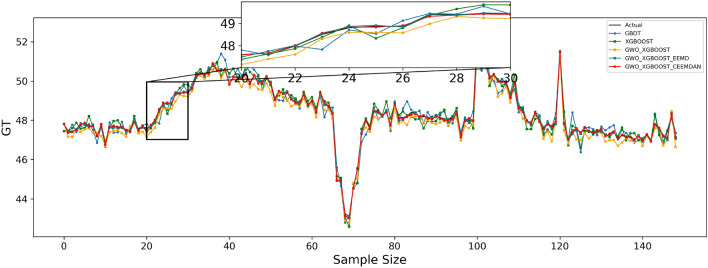
Hubei model prediction effect.

**Figure 6:**
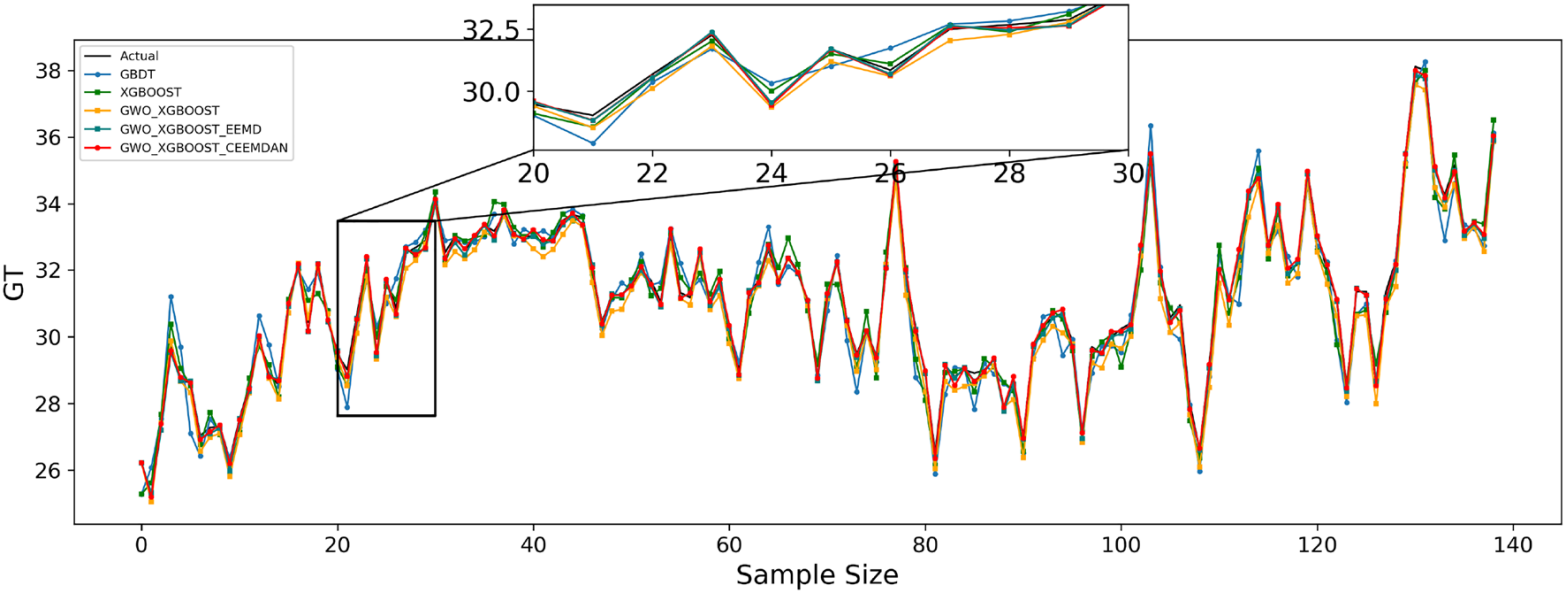
Fujian model prediction effect.

Table 9 shows the effect of the fitted curves on the carbon price predictions for each model in the three test sets. Furthermore, to facilitate a visual comparison of the prediction outcomes between the proposed model and other comparative models, Figs. 4, 5, and 6 depict the prediction results for the Guangdong, Hubei, and Fujian datasets, respectively.

**Table 9:** Algorithm experiment results table.

Forecast range	Evaluation indicators	GBDT	XGBOOST	GWO–XGBOOST	GWO–XGBOOST–EEMD	GWO–XGBOOST–CEEMDAN
Guangdong	MSE	0.650193	0.410348	0.368359	0.119977	**0.078120**
	MAE	0.508363	0.391618	0.436538	0.275044	**0.223989**
	RMSPE	1.013249	0.806493	0.775963	0.441638	**0.356462**
	MAPE	0.642882	0.495045	0.556786	0.003506	**0.002856**
	R^2^	0.931976	0.957069	0.961462	0.9874478	**0.991826**
Hubei	MSE	0.101325	0.081960	0.075187	0.005043	**0.004184**
	MAE	0.240302	0.216567	0.232021	0.056902	**0.050459**
	RMSPE	0.657276	0.589355	0.567327	0.147217	**0.134632**
	MAPE	0.496693	0.447754	0.480307	0.001180	**0.001047**
	R^2^	0.949081	0.958812	0.962216	0.997466	**0.997898**
Fujian	MSE	0.299317	0.170274	0.075187	0.005043	**0.004184**
	MAE	0.407047	0.332075	0.232021	0.056902	**0.050459**
	RMSPE	1.802013	1.347809	0.567327	0.147217	**0.134632**
	MAPE	1.328623	1.080592	0.480307	0.001180	**0.001047**
	R^2^	0.945945	0.958812	0.962216	0.997466	**0.997898**

Significant values are given in Bold.

### Experiment two: comparison of this paper’s model with different input feature models

#### Algorithm table

To further confirm the effectiveness of the feature selection algorithm, GWO–XGBOOST–CEEMDAN*, All-VARIABLE-GWO–XGBOOST–CEEMDAN, and the proposed GWO–XGBOOST–CEEMDAN model are compared in this paper, and the algorithm experiment table is shown in Table 10.

**Table 10:** Algorithm experiment table.

Models	External influencing factors	Historical carbon price
GWO–XGBOOST–CEEMDAN*		**√**
ALL-VARIABLE- GWO–XGBOOST–CEEMDAN	**√**	**√**
PROPOSED MODEL	**√**	**√**

#### Carbon price forecast results II

**Figure 7:**
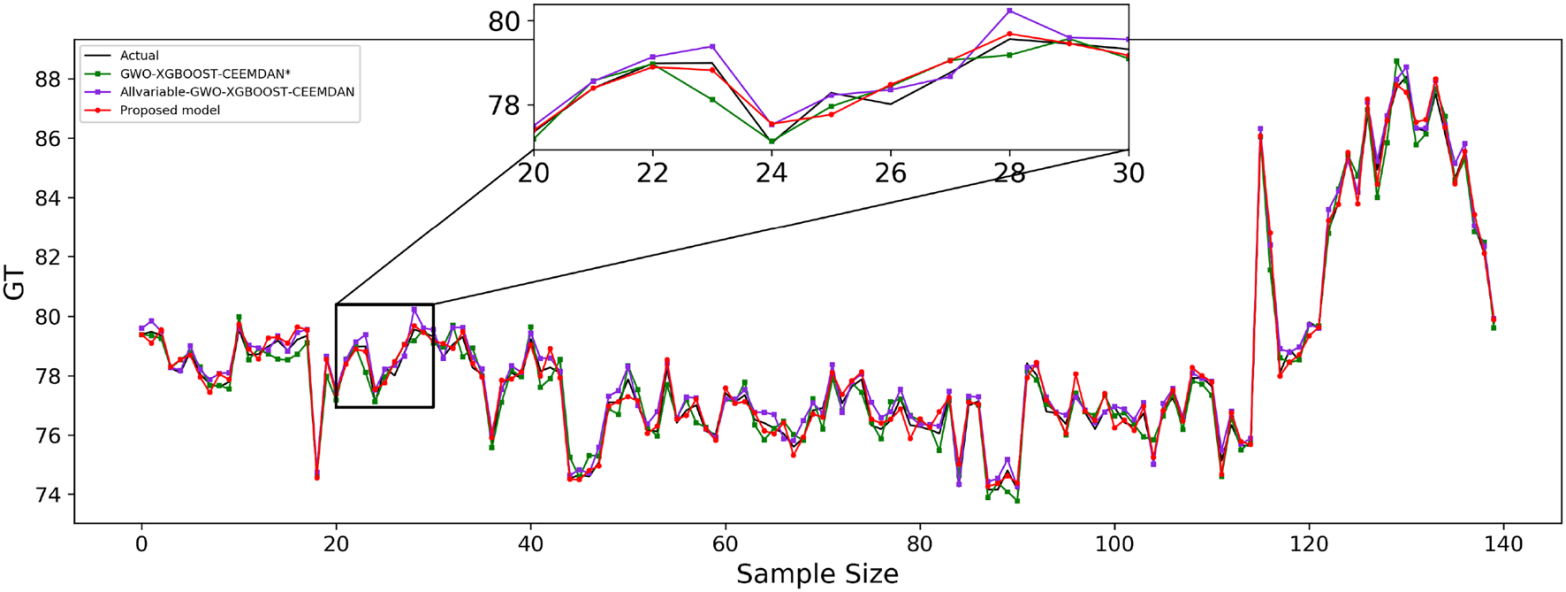
Guangdong model prediction effect.

**Figure 8:**
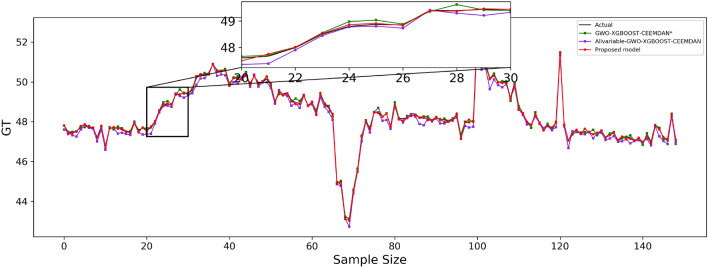
Hubei model prediction effect.

**Figure 9:**
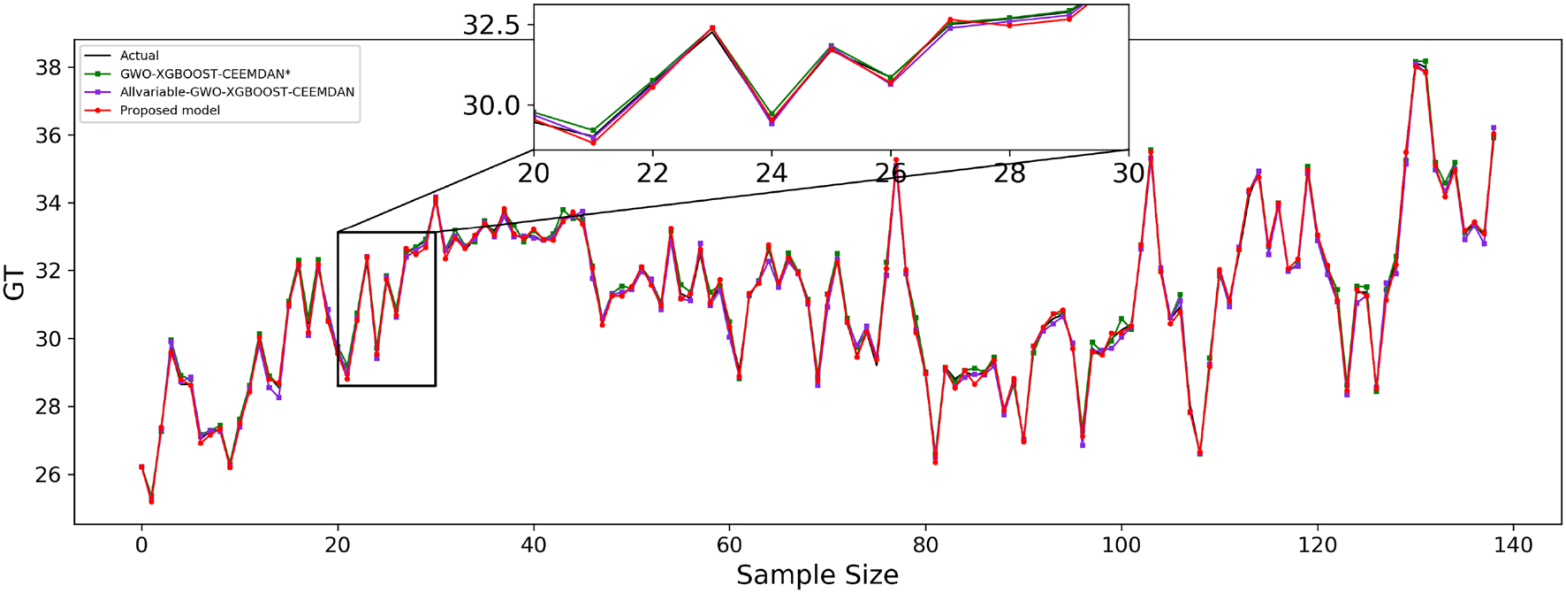
Fujian model prediction effect.

The predicted effect graphs are shown in Figs. 7, 8, and 9, and the algorithm experimental results table is shown in Table 11.

**Table 11:** Algorithm experiment results table.

Forecast range	Evaluation indicators	GWO–XGBOOST–CEEMDAN*	ALL-VARIABLE- GWO–XGBOOST–CEEMDAN	PROPOSED MODEL
Guangdong	MSE	0.142878	0.147749	**0.078120**
	MAE	0.302748	0.307266	**0.223989**
	RMSPE	0.479748	0.488676	**0.356462**
	MAPE	0.003858	0.003911	**0.002856**
	R^2^	0.985052	0.984542	**0.991826**
Hubei	MSE	0.006618	0.029784	**0.004184**
	MAE	0.065209	0.139503	**0.050459**
	RMSPE	0.168078	0.357332	**0.134632**
	MAPE	0.001348	0.002888	**0.001047**
	R^2^	0.996674	0.985033	**0.997898**
Fujian	MSE	0.020320	0.021822	**0.013272**
	MAE	0.114830	0.119552	**0.095404**
	RMSPE	0.462370	0.475852	**0.371209**
	MAPE	0.003709	0.003846	**0.003070**
	R^2^	0.996330	0.996059	**0.997603**

Significant values are given in Bold.

### Analysis of experimental results

**Figure 10:**
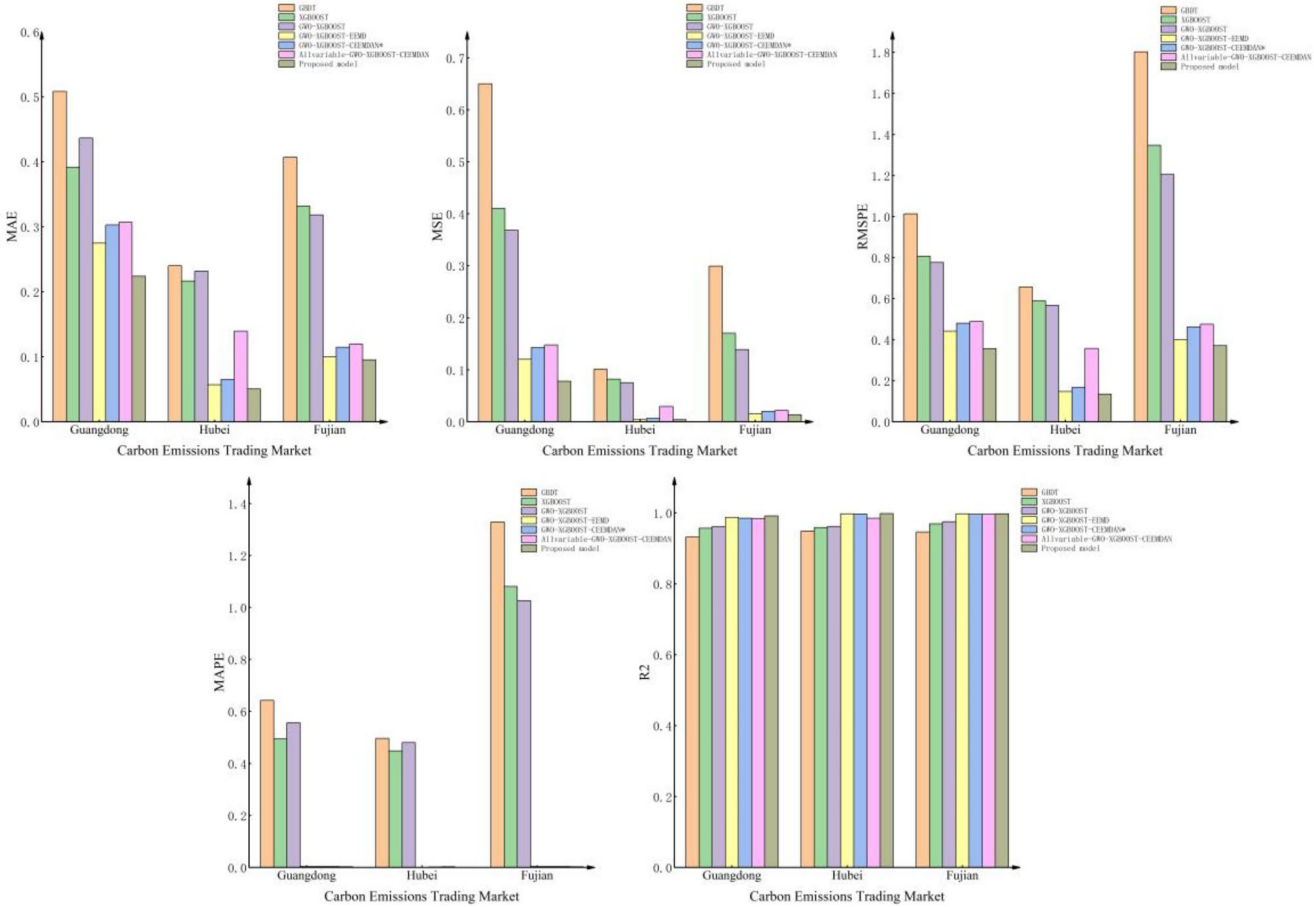
Results of index evaluation of each model.

Figure 10 shows the predicted results of the evaluation indexes of the above two groups of experiments. By comparing and analyzing the above two sets of experiments, we can draw the following conclusions:

1. In single-model prediction, the GWO–XGBOOST model has the best prediction effect, which is mainly attributed to the following reasons: First, GBDT builds an integrated model by training a series of decision trees; each tree is trained on the residuals of the previous tree, so when the number of trees is large, the model may be over-fitted on the training set, resulting in large model prediction errors. Second, XGBOOST is an integrated learning method that enhances and optimizes on the basis of GBDT and improves generalization ability but has weaker performance in dealing with the category imbalance problem. Finally, GWO, as an optimization algorithm for searching for the global optimal solution, can not only tune the hyperparameters in XGBOOST, such as learning rate, tree depth, subsample ratio, etc., but also fuse multiple XGBOOST models, adjusting the weights and parameters of different models to achieve the combination and integration of models. This can, to some degree, enhance the model’s stability and generalization ability, thus increasing its overall capability.
2. For the combination algorithm, all combination models outperform comparison models in relation to predictive accuracy. The Guangdong dataset is used as an example, and the Hubei and Fujian datasets are consistent with this conclusion. First, to verify the effectiveness of the decomposition method proposed in this paper, GWO–XGBOOST–EEMD is contrasted with the model in this paper, and it is found that the prediction accuracy of the proposed model in this paper is improved by 34.888%, 18.562%, 19.286%, 18.540%, and 0.443% for MSE, MAE, RMSPE, MAPE, and R^2^, respectively. The results indicate that the CEEMDAN approach proposed in this study offers an additional enhancement to the prediction accuracy of the GWO–XGBOOST model when compared to EEMD. Secondly, to further demonstrate the superiority of the feature selection algorithm, the GWO–XGBOOST–CEEMDAN* model only considers carbon price historical data, and the All-VARIABLE-GWO–XGBOOST–CEEMDAN model takes carbon price historical data and all external influences as input variables. The results of these three models indicate that the feature selection algorithm is helpful in improving the prediction performance of the hybrid model.

### Discussion

#### Validation on other data sets

In order to verify the generalization ability of the model as well as its strong robustness, we selected a Q1 partitioned article published in the journal ENVIRONMENTAL SCIENCE AND POLLUTION RESEARCH^29^. This paper selects the daily carbon prices in Beijing, Hubei, and Shanghai as sample data, all of which come from the China Carbon Trading Network, and the experiment uses 75% of the data as the training set and 25% as the test set as a way to verify the accuracy of the hybrid model. We use the raw data from this published paper to do a comparative analysis between our proposed model and the model prediction results from the published paper, which not only verifies again that our own model has strong stability and prediction accuracy but also makes the study richer and more convincing. The information on the three carbon trading markets is shown in Table 12.

**Table 12:** Carbon trading market data information.

Market	Dataset	Training set	Test set	Training date	Test date	Time nodes
Beijing	1577	1183	394	2013.11.28–2018/9/7	2018/9/10–2020.4.27	2013.11.28–2020.4.27
Hubei	1525	1144	381	2014.4.3–2018/9/27	2018/9/28–2020.4.27	2014.4.3–2020.4.27
Shanghai	1561	1171	390	2013.12.19–2017/4/19	2017/4/20–2020.4.27	2013.12.19–2020.4.27

**Figure 11:**
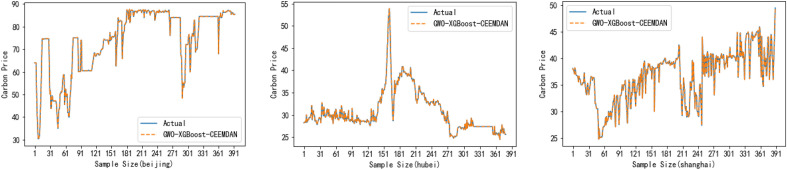
Model prediction effect.

The effect of the fitting curves of our carbon price prediction for three carbon markets using the proposed GWO–XGBOOST–CEEMDAN model is shown in Fig. 11. In addition, Table 13 is a table of the experimental results of the algorithms of the two models for the three carbon markets, which compares more intuitively the prediction accuracy capability of the proposed model with the models of the published papers.

**Table 13:** Algorithm experiment results table.

Forecast range	Model	MAE	MAPE	RMSE
Beijing	Published paper model	2.543	0.232	3.363
	Proposed model	**0.0847**	**0.0012**	**0.7177**
Hubei	Published paper model	0.654	0.144	1.048
	Proposed model	**0.0265**	**0.0009**	**0.0328**
Shanghai	Published paper model	1.212	0.143	1.673
	Proposed model	**0.0212**	**0.0006**	**0.0271**

Significant values are given in Bold.

Comparing the prediction accuracy of our own proposed model with the published paper model for the same dataset, we can draw the following conclusions:

1. Most of the previous research focused on the analysis of historical carbon price prediction, and the selected published paper is a typical representative of the previous research, which is different from one of the innovations of my own thesis: considering the historical carbon price and the influencing factors at the same time so as to create a rich indicator system. Therefore, this published paper also validates, to a certain extent, the prediction accuracy of the proposed model when only considering the historical carbon price.
2. The research objects of the published papers are selected as Beijing, Hubei, and Shanghai, which are different from the objects of our own paper. Firstly, it verifies that the proposed model is not limited to the carbon market in Guangdong, Hubei, and Fujian but can also be used in carbon price prediction studies in other regions, which means that the results can be extended to other regions, such as Beijing and Shanghai. Secondly, the research object in the published paper still includes the Hubei carbon market, which verifies that the proposed model still has high accuracy in the case of another dataset that only considers the historical carbon price. In conclusion, by comparing the carbon price prediction with the published paper for the same dataset, it shows that the proposed model has stronger generalization ability and robustness.
3. The GWO–XGBOOST–CEEMDAN model is more suitable than the VMD–SE–DRNN–GRU model used in the published paper to deal with the problem of forecasting time series data. Firstly, CEEMDAN automatically determines the number of modes to be generated based on the dataset and generates intrinsic modal functions (IMFs), while VMD, although it can also perform modal decomposition, needs to pre-specify the number of modes to be decomposed into, which requires some domain knowledge or experiments to determine. If the number of modes chosen is inappropriate, it may lead to inaccurate decomposition results, thus CEEMDAN has more adaptive and flexible compared to VMD. Secondly, the GWO–XGBOOST–CEEMDAN model has lower complexity compared to deep learning models, thus it is easier to train with limited data and does not require a large amount of computational resources, which can make it more practical in some applications, such as the field of carbon price prediction. Finally, XGBOOST models are usually very interpretable and can provide feature importance rankings, whereas deep learning models such as DRNN and GRU are usually more difficult to interpret, especially in highly complex network structures. In conclusion, both VMD–SE–DRNN–GRU and GWO–XGBOOST–CEEMDAN are sophisticated carbon price prediction methods, and GWO–XGBOOST–CEEMDAN may be a better choice in cases of complex or non-stationary data.

#### DM test

To further examine the prediction performance between the proposed GWO–XGBOOST–CEEMDAN integrated combination model and the comparison models, this section uses the DM test to analyze statistical errors from the perspective of statistical errors. The bold values in the table indicate that the *p*-value is below the significance threshold of 0.05. To visually assess the predictive performance of the GWO–XGBOOST–CEEMDAN model and other models, we analyze their predictive ability using the coverage ratio based on the DM results. The coverage ratio is expressed as the ratio of the number of DM results rejecting the original hypothesis to the total number of DM results. When the models exhibit comparable predictive capabilities, a lower number of DM test results have a *p*-value less than 0.05, resulting in a coverage rate below 50%. When the models demonstrate significantly superior predictive capabilities compared to the benchmark model, a higher number of DM test results exhibit a *p*-value below 0.05, leading to a coverage rate exceeding 50%.

This further analysis of the DM test results in Table 14 revealed that DM coverage for the GWO–XGBOOST–CEEMDAN model was 83.3% in all three datasets, demonstrating that the GWO–XGBOOST–CEEMDAN model outperformed the proposed benchmark model in the majority of instances, and thus the proposed hybrid model was statistically significant.

**Table 14:** DM test results.

Models	Guangdong		Hubei		Fujian	
	MAE	MSE	MAE	MSE	MAE	MSE
GBDT	**1.31480e-07**	**0.000140**	**0.000000**	**2.31306e-10**	**0.000000**	**3.09520e-10**
XGBOOST	**0.000100**	**0.002870**	**0.000000**	**3.12004e-10**	**0.000000**	**4.68075e-11**
GWO-XGBOOST	**3.02359e-09**	**7.92154e-06**	**0.000000**	**0.000000**	**0.000000**	**0.000000**
GWO-XGBOOST-EEMD	**0.015140**	**0.009720**	0.130250	0.215960	0.464508	0.254400
GWO-XGBOOST-CEEMDAN*	**0.000610**	**0.000350**	**0.003640**	**0.007930**	**0.033014**	**0.009580**
All-VARIABLE-GWO-XGBOOST-CEEMDAN	0.411980	0.632980	**0.000000**	**1.04716e-12**	**0.006089**	**0.001100**

a *p*-value of 0.0 does not mean that the data is 0, it just means that the *p*-value is small and the probability tends to be 0.

#### Limitations of the current study and future work

Although the constructed hybrid forecasting framework showcases superior performance in carbon trading price prediction and fills the current research gap in carbon price prediction, there are still a few shortcomings that need further improvement and development. Following are the main limitations of this study:

1. Due to data availability limitations, the hybrid prediction framework we developed only considers eight influencing factors.
2. This study provides information for related scholars. Firstly, this paper performs carbon price prediction first and then decomposes the residual series. As a result, data can be explored and utilized more effectively, and prediction accuracy and reliability can be improved. Compared with the traditional method of decomposition followed by prediction, this paper provides an alternative way of thinking and method for carbon price prediction, which scholars can refer to further to explore and analyze the intrinsic mechanism of carbon price, expand the research field, and deepen theoretical understanding. Second, in the modeling process, in addition to the factors already considered, other factors that may affect carbon prices can be further considered. This will reveal more factors affecting carbon price change and further improve the prediction effect. In addition to better adapting to changes in data characteristics, we need to improve the hybrid forecasting framework. Specifically, we can implement automatic key parameter settings and build a smart carbon price prediction framework. This framework automatically adapts to data changes, improving prediction accuracy and stability. Finally, future research can also extend carbon price prediction to other fields, for instance, energy market prediction and climate change risk management, which will further prove the value of our research results.

#### Impact on sustainability

This study examines the effects of macroeconomics, energy prices, international carbon markets, and weather conditions on improving carbon trading price forecasts, which have crucial ramifications for sustainable development. In particular, risk managers can incorporate multiple factors, such as energy factors and global carbon prices, into carbon market management. In addition, investors can grasp carbon market dynamics based on influencing factors and improve market participants’ flexibility and motivation. This paper conducts research related to carbon price forecasting, which is helpful for the government and enterprises to grasp the characteristics of carbon prices and helps carbon market management and investment decisions, especially the solution of the carbon price prediction problem, which is linked to whether the double carbon target can be achieved on time or in advance. Therefore, this study aims to provide a reasonable forecast of carbon prices in order to facilitate carbon market participants in achieving their goals and help real producers reduce emissions efficiently through market mechanisms. In conclusion, the forecasting framework and the associated research findings we have developed hold significant implications for the advancement of sustainable development.

#### Feature importance analysis

**Figure 12:**
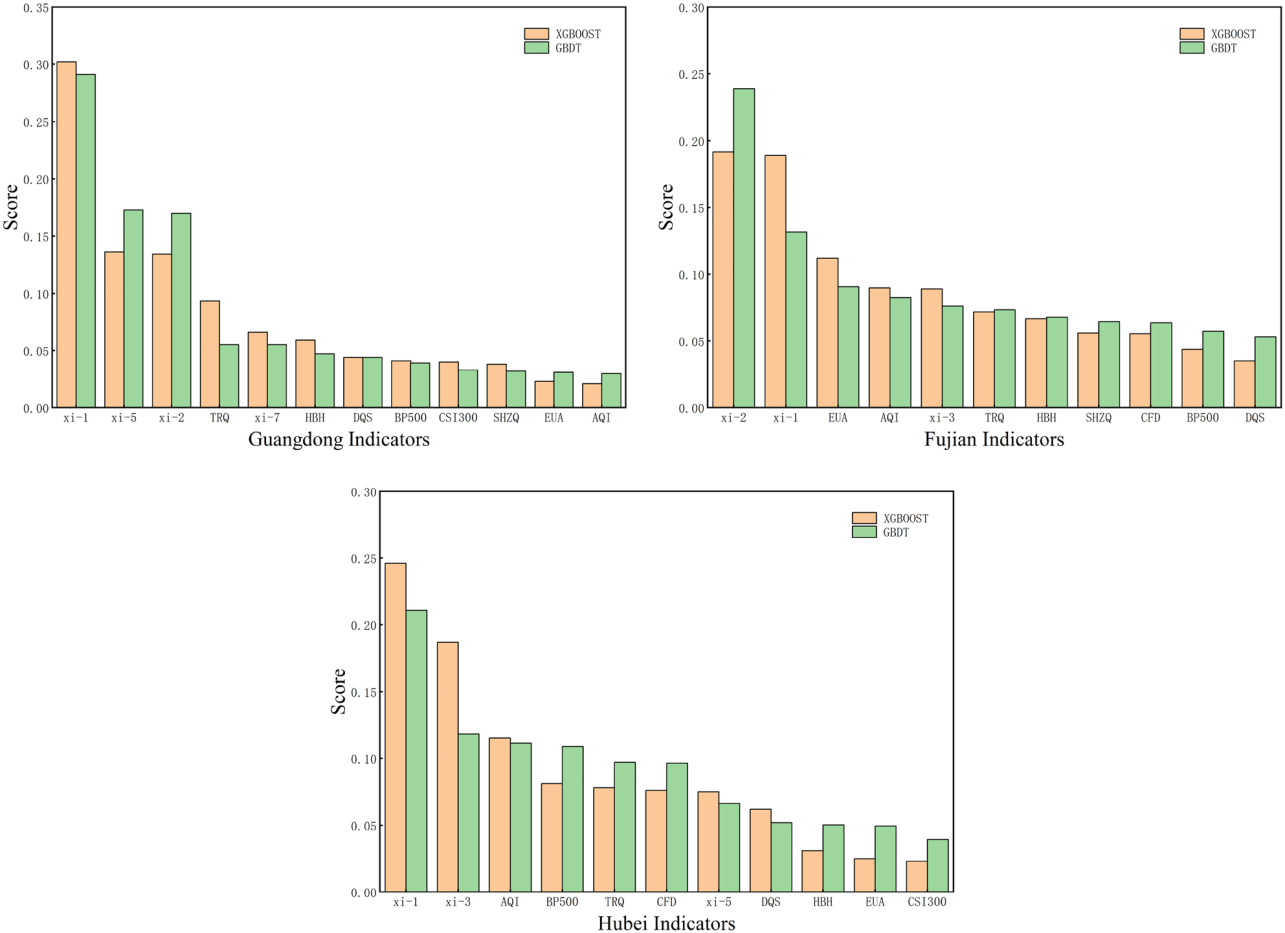
Feature importance diagram.

To identify the key determinants in carbon price prediction, this paper uses XGBOOST and GBDT models for feature importance analysis, respectively. The results and statistical plots of feature importance indices for each model are shown in Table 13 and Fig. 12. Through preliminary observation, it is evident that the feature ranking results in the two models for the three data sets vary to some extent. A more in-depth examination of the feature rankings in Table 15 uncovers that the historical carbon price, natural gas market offers, and Bohai Ring Power Coal Price Index in energy prices, the S&P 500 and Dow Jones Industrial Index in macroeconomics, and the EU carbon emission allowances in the international carbon market rank ahead of the two models for the three data sets XGBOOST and GBDT as the key factors for carbon price prediction.

**Table 15:** Carbon price characteristics in order of importance.

Guangdong				Hubei				Fujian			
XGBOOST		GBDT		XGBOOST		GBDT		XGBOOST		GBDT	
Features	Score	Features	Score	Features	Score	Features	Score	Features	Score	Features	Score
xi-1	0.302	xi-1	0.291	xi-1	0.246	xi-1	0.211	xi-2	0.192	xi-1	0.239
xi-5	0.136	xi-5	0.173	xi-3	0.187	xi-3	0.118	xi-1	0.189	xi-3	0.132
xi-2	0.134	xi-2	0.170	AQI	0.115	xi-5	0.111	EUA	0.112	DQS	0.091
TRQ	0.093	xi-7	0.055	BP500	0.081	TRQ	0.109	AQI	0.090	BP500	0.083
xi-7	0.066	EUA	0.055	TRQ	0.078	CSI300	0.097	xi-3	0.089	EUA	0.076
HBH	0.059	BP500	0.047	CFD	0.076	EUA	0.096	TRQ	0.072	SHZQ	0.073
DQS	0.044	DQS	0.044	xi-5	0.075	CFD	0.066	HBH	0.067	xi-2	0.068
BP500	0.041	TRQ	0.039	DQS	0.062	HBH	0.052	SHZQ	0.056	CFD	0.065
CSI300	0.040	HBH	0.033	HBH	0.031	DQS	0.050	CFD	0.056	HBH	0.064
SHZQ	0.038	AQI	0.032	EUA	0.025	AQI	0.050	BP500	0.044	TRQ	0.057
EUA	0.023	CSI300	0.031	CSI300	0.023	BP500	0.039	DQS	0.035	AQI	0.053
AQI	0.021	SHZQ	0.030								

For governments, our findings suggest that historical carbon prices, natural gas market quotes, the Bohai Ring Power Coal Index, the S&P 500 Index, the Dow Jones Industrial Average, and EU carbon emission allowances can be effective ways to improve the predictive power of carbon prices in regional carbon trading markets, and policymakers can refer to our findings to make decisions about carbon market policies. First, for historical carbon price data, when the carbon price rises, the government makes stricter carbon reduction policies. This is to stimulate emission reduction measures. And when carbon prices fall, the government may reduce subsidies and support for carbon abatement to prevent a burden on the Treasury. Second, changes in natural gas supply and the Bohai Ring Power Coal Index can affect energy security. When these prices rise, the government can increase domestic production and reserves to guarantee energy supply stability. When prices fall, the government should promote the energy market by increasing subsidies and controlling imports. Moreover, the S&P 500 and the Dow Jones Industrial Average reflect the macroeconomic environment. When prices fall, the government takes stimulus measures, such as cutting taxes or increasing spending, to promote economic growth. When prices rise, the government can take restraining measures, such as strengthening regulation or controlling capital inflows, to prevent overheating. Finally, EU carbon emission allowances reflect international carbon markets. When quotas rise, the government should increase carbon emission quotas to ease enterprises’ economic burden. This will avoid excessive carbon prices that lose them competitiveness. When the quota decreases, the government should reduce carbon emission quotas or support low-carbon technologies to reduce carbon emissions. In addition, the government should strengthen regulation and management to reduce fraud and misconduct on the carbon market, which will improve market transparency and stability.

In conclusion, the carbon price forecasting study in this paper can act as a point of reference for policymakers to consider various factors. This will ensure policy sustainability and effectiveness.

## Conclusions

This paper uses sophisticated data decomposition methods and efficient feature subset selection algorithms to propose a comprehensive consideration of multiple influencing factors in a carbon price forecasting framework, thus achieving the expected forecasting results. To validate the effectiveness of the designed hybrid forecasting framework, we conducted an empirical study on three regional carbon emission trading markets in Guangdong Province, Hubei Province, and Fujian Province, China, and evaluated five performance evaluation indicators, six benchmark models, three case analyses, and four discussions to systematically and holistically examine the developed hybrid forecasting framework. The prediction results clearly demonstrate the superior performance of the developed prediction framework over all benchmark models. Therefore, we contend that the proposed carbon price forecasting framework offers a valuable and effective approach to predicting carbon prices. Specifically, this study’s findings can be briefly outlined as follows:

1. The XGBOOST model based on the boosting integrated learning framework has high prediction accuracy and strong generalization ability. However, the model’s predictive performance is sensitive to parameter settings, and the grey wolf optimization algorithm facilitates rapid determination of the optimal parameters for the XGBOOST model. Among the single-model carbon price prediction approaches, the proposed GWO–XGBOOST model exhibits the highest level of accuracy.
2. The nonlinear residual series generated by an individual machine learning model for carbon price prediction still contains valid information. In this paper, CEEMDAN is utilized to decompose the residual series generated by the GWO–XGBOOST model into sub-series with different frequencies, so that each sub-series is predicted separately, and finally, the prediction results of each sub-series are superimposed to achieve the overall prediction result. In comparison to the traditional single model, the combined GWO–XGBOOST–CEEMDAN model presented in this paper has the most accurate prediction effect.
3. Two machine learning models, XGBOOST and GBDT, are used to model carbon prices separately and perform feature importance analyses. It is found that the historical carbon price, the natural gas market price, and the Bohai Sea Power Coal Price Index in the energy price, the macroeconomic S&P 500 and Dow Jones Industrial Average in the macroeconomy, and EU carbon emission allowances in the international carbon market are the main influencing factors for carbon price prediction.
4. This study constructs a carbon price prediction model in accordance with multiple influencing factors and introduces a feature selection method. By selecting the features with the most predictive power from many possible input features and mitigating the adverse impacts of redundant information and noise among features, the model’s predictive performance and generalization capability are enhanced.
5. In this paper, external influences and historical carbon price data are jointly used as input features of the model, such as macroeconomics, energy prices, and international carbon markets. Compared with previous studies that only considered historical carbon price data and all external influences and historical carbon price data concurrently, the accuracy of carbon price prediction has significantly improved. For one thing, it shows the significance of feature selection, and for another, it indicates that the combination of external factors and historical carbon price data can forecast the carbon price trend more precisely.

## Data Availability

The data used to support the findings of this study are available from the corresponding author upon request.
